# Cat-Scratch Disease Associated with Acute Hearing Loss, Israel

**DOI:** 10.3201/eid3207.260592

**Published:** 2026-07

**Authors:** Michal Yakubovsky, Michal Katzir, Alaa Atamna, Dana Yelin, Michal Landes, Gabriel Weber, Moshe Ephros, Michael Giladi

**Affiliations:** Tel Aviv University, Gray Faculty of Medical and Health Sciences, Tel Aviv, Israel (M. Yakubovsky, M. Katzir, A. Atamna, D. Yelin, M. Giladi); Tel Aviv Sourasky Medical Center, Tel Aviv (M. Yakubovsky, M. Giladi); Meir Hospital, Kfar Saba, Israel (M. Katzir); Beilinson Hospital, Rabin Medical Center, Petach Tiqva, Israel (A. Atamna); Sheba Medical Center, Ramat Gan, Israel (D. Yelin); Technion–Israel Institute of Technology, Rappaport Faculty of Medicine, Haifa, Israel (M. Landes, G. Weber, M. Ephros); Emek Medical Center–Clalit, Afula, Israel (M. Landes); Carmel Medical Center, Haifa (G. Weber)

**Keywords:** cat-scratch disease, Bartonella henselae, bacteria, zoonoses, hearing loss, sudden, sensorineural, Israel

## Abstract

We report 5 patients in Israel with cat-scratch disease (CSD) who had unilateral sudden sensorineural hearing loss. Several mechanisms are plausible, but further research is needed to clarify pathogenesis. The cases highlight a previously underrecognized association between CSD and sudden sensorineural hearing loss, expanding the spectrum of cranial nerve neuropathies in CSD.

Cat-scratch disease (CSD), caused by *Bartonella henselae* bacteria, is a zoonotic infection primarily transmitted through contact with cats, mainly scratches or bites. Approximately 90% of cases, designated as typical CSD, are characterized by regional lymphadenitis. Atypical CSD manifestations are diverse and include fever of unknown origin (FUO), erythema nodosum, hepatosplenic involvement, neuroretinitis, and neurologic complications such as encephalitis, and cranial nerve neuropathy (*1*,*2*). Sudden sensorineural hearing loss (SSNHL) has been associated with viral and bacterial infections (*3*), but it is not a recognized sequela of CSD.

On the basis of data from a national CSD registry in Israel with long-term follow-up, we report 5 patients with CSD who had SSNHL. This case series suggests a potentially underrecognized association between CSD and SSNHL.

## The Study

We have conducted a surveillance study of CSD in Israel since 1991 (*4*). We defined CSD as illness in a patient with symptoms and signs consistent with CSD in the absence of another diagnosis and >1 confirmatory laboratory result: a positive serologic test for *B. henselae* antibodies (IgM, IgG, or both) or a positive PCR for *B. henselae* DNA. We performed serologic testing by using enzyme immunoassay (EIA) and interpreted results as previously described (*5*). We performed PCR on lymph node tissue as previously reported (*4*). We confirmed SSNHL by audiometry when the patient was admitted for care. Clinical outcome was based on patient-reported hearing because repeat audiometric testing was not available. We considered doxycycline, macrolides, and rifampin to be active against *B. henselae*. The Tel Aviv Sourasky Medical Center Institutional Review Board approved the study (approval no. TLV-0147-08). We collected follow-up data prospectively and obtained informed consent from all patients.

Five patients with CSD had unilateral SSNHL, confirmed by audiometric testing performed after an otolaryngologic evaluation. Two patients also reported tinnitus. All patients had >1 additional manifestations characteristic of CSD, including FUO (4/5), lymphadenitis (3/5), ocular CSD manifestations (3/5), erythema nodosum (1/5), and encephalitis (1/5). Among the 4 patients with FUO, fever was continuous or intermittent, lasting 3–12 weeks (median 6 weeks). The fifth patient had no fever but reported prolonged weakness (Table).

**Table:** Characteristics of CSD patients with sudden sensoneuronal hearing loss, Israel*

Characteristic	Patient no.				
	1	2	3	4	5
Age, y	35	47	71	15	35
Sex	Female	Female	Male	Female	Male
Cat contact	Yes	Yes	Yes	Yes	Yes
Fever (duration, wks)	Yes (8)	Yes (3)	Yes (4)	No	Yes (12)
Lymphadenopathy	Not reported	Not reported	Retroperitoneal	Retro-auricular	Cervical
Atypical CSD manifestations	Neuroretinitis, CSD-FUO syndrome	Central retinal artery occlusion, chorioretinitis, CSD-FUO syndrome	CSD-FUO syndrome	Neuroretinitis	Erythema nodosum, encephalitis, CSD-FUO syndrome
Time from CSD onset to hearing loss, wks	9	3	5	6	52
Laboratory diagnosis of CSD	IgG 1:200; IgM positive	IgG 1:800; IgM negative	IgG 1:400; IgM negative	IgG 1:100 (IgG seroconversion); IgM negative; positive PCR (lymph node)	IgG 1:200; IgM borderline
Antibiotics	Doxycycline and rifampin	Doxycycline	None	None	Doxycycline and rifampin
Corticosteroids	Yes	Yes	Yes	No	No
Outcome	Partial recovery with residual hearing impairment	Partial recovery with residual hearing impairment	Recovery after 4 wks	Recovery after 10 wks	Partial recovery with residual hearing impairment
Duration of follow-up, mo	107	75	58	85	29

*CSD, cat-scratch disease; FUO, fever of unknown origin. †Based on the patients’ own subjective perception of hearing ability because repeat audiometric testing was not available. For complete recovery, duration of hearing loss is shown; for partial recovery, hearing was assessed at last follow-up.

In 4 patients, SSNHL occurred 3–9 weeks after initial CSD diagnosis. The fifth patient, a musician, experienced severe, prolonged CSD, beginning with 3 months of intermittent fever and unilateral cervical lymphadenitis, during which he had onset of encephalitis eventually requiring hospitalization, followed by persistent fatigue. SSNHL occurred 9 months after CSD diagnosis. At the last follow-up, 29 months after SSNHL onset, he reported persistent hearing impairment but declined further evaluation.

Three patients received relevant antibiotics (none received azithromycin), 3 received oral corticosteroids, and 3 received no treatment (Table). After a median follow-up of 66 months (range 29–107 months), 3 (60%) of 5 patients reported partial recovery, with residual hearing impairment, and 2 reported complete recovery.

In this case series, we report 5 patients with CSD and SSNHL. A temporal association between active CSD and SSNHL was evident in 4 patients. In contrast, patient 5 had hearing loss 1 year after the onset of a prolonged CSD, making causality less certain.

SSNHL is a recognized but uncommon sequela of viral and bacterial infections (*3*). Our literature search identified only 2 early reports predating the identification of *B. henselae* as the etiologic agent of CSD in 1992 (*6*,*7*), suggesting this association may be underrecognized.

Although the diagnosis of CSD often is challenging, it was well supported in our patients by epidemiologic, clinical, and laboratory data: all patients reported cat contact and exhibited various CSD manifestations, including lymphadenopathy, FUO, encephalitis, erythema nodosum, and ocular findings (*1*), and all patients had serologic confirmation (Table). Of note, serodiagnosis of CSD usually is IgG-based, whereas IgM is short-lived and detectable in approximately half of CSD patients (*5*).

Ancillary testing for alternative infections (e.g., HIV and syphilis) was inconsistent and generally performed for other indications (e.g., prolonged fever), rather than the hearing loss. This approach aligns with current guidelines, which do not recommend routine laboratory testing in SSNHL because of limited yield (*8*). All cases predated COVID-19, and Lyme disease is not endemic in Israel; therefore, corresponding laboratory testing was not performed.

The pathogenesis of SSNHL in CSD remains unknown. In viral-associated SSNHL, such as herpes simplex, mumps, measles, rubella, HIV, and enteroviruses, proposed mechanisms include direct cochlear invasion, reactivation of latent virus, and immune-mediated injury (*9*). Similar processes have been suggested in COVID-19–associated hearing loss, and imaging findings supported cochlear inflammation and occasional nerve involvement (*9*). In bacterial infections, mechanisms are less defined. *Rickettsia* spp., known to be intracellular and endotheliotropic like *B. henselae*, have been associated with hearing loss, possibly mediated by immune-related vasculitic involvement of the cochlear or cochlear nerve vasculature (*10*).

Several observations in *Bartonella* infection provide biologic plausibility for the proposed mechanisms underlying SSNHL. In vitro studies show that *B. henselae* can infect feline microglial brain cells (*11*). Animal studies further indicate dissemination to the central nervous system (CNS); in a feline model, *B. henselae* was recovered from brain tissue after intradermal inoculation (*12*), consistent with the potential for neural involvement. Clinical observations further support cranial nerve involvement and potential pathogenic mechanisms. *B. henselae* has been identified in human CNS tissue through autopsy, indicating direct invasion of neural structures (*13*). In addition, the endothelial tropism of *B. henselae* is evidenced by its association with vascular injury, including cerebral vasculitis, in human brain biopsy (*14*). Moreover, retinal vascular occlusion and ischemic optic neuropathy in CSD patients (*1*) suggest that microvascular injury might contribute to dysfunction of the cochlear or cochlear nerve, structures known to be highly vulnerable to ischemia. Furthermore, a pediatric case of CSD-associated peripheral facial nerve palsy demonstrated a granulomatous lesion at the internal auditory meatus that resolved with treatment (*15*). Given the intimate anatomic proximity of cranial nerves VII and VIII within this compartment, a similar inflammatory lesion could affect the cochlear nerve, causing neuritis, compressive neuropathy, or both. Our recent report of multiple cranial neuropathies in CSD, involving cranial nerve III, VI, VII, and IX (*2*), further supports the potential for *B. henselae* infection to involve cranial nerves and cause neuropathic manifestations. Immune-mediated inflammatory mechanisms could also contribute, although current evidence remains indirect (*3*).

Taken together, data support 3 non–mutually exclusive mechanisms for SSNHL in CSD: vascular or vasculitic injury of the cochlea or cochlear nerve, analogous to rickettsial disease; focal inflammatory involvement of the vestibulocochlear nerve within the internal auditory canal, potentially causing neuritis, compressive neuropathy, or both within this anatomically confined space; and immune-mediated cranial neuritis occurring in the context of systemic *Bartonella* infection. Although definitive proof is lacking, the ability of *B. henselae* to involve neural and neurovascular components of the CNS, together with its recognized associations with cranial neuropathies, provides a biologically plausible explanation for transient nerve VIII dysfunction and sudden hearing loss.

The small number of cases and the heterogeneity of treatment preclude therapeutic conclusions. Of note, none of the patients received azithromycin, excluding azithromycin-associated SSNHL as a confounder.

## Conclusions

We report a possible but underrecognized association between CSD and SSNHL that warrants further clinical awareness. Several mechanisms are plausible, but further research is needed to clarify the pathogenesis. All 5 patients had additional CSD manifestations, which, together with the sudden hearing loss, posed a diagnostic challenge. A history of cat exposure may serve as a critical clue prompting appropriate laboratory testing. 
